# A comprehensive framework for advanced protein classification and function prediction using synergistic approaches: Integrating bispectral analysis, machine learning, and deep learning

**DOI:** 10.1371/journal.pone.0295805

**Published:** 2023-12-14

**Authors:** Hiam Alquran, Amjed Al Fahoum, Ala’a Zyout, Isam Abu Qasmieh

**Affiliations:** Hijjawi Faculty for Engineering Technology, Biomedical Systems and Informatics Engineering Department, Yarmouk University, Irbid, Jordan; University of Manitoba, CANADA

## Abstract

Proteins are fundamental components of diverse cellular systems and play crucial roles in a variety of disease processes. Consequently, it is crucial to comprehend their structure, function, and intricate interconnections. Classifying proteins into families or groups with comparable structural and functional characteristics is a crucial aspect of this comprehension. This classification is crucial for evolutionary research, predicting protein function, and identifying potential therapeutic targets. Sequence alignment and structure-based alignment are frequently ineffective techniques for identifying protein families.This study addresses the need for a more efficient and accurate technique for feature extraction and protein classification. The research proposes a novel method that integrates bispectrum characteristics, deep learning techniques, and machine learning algorithms to overcome the limitations of conventional methods. The proposed method uses numbers to represent protein sequences, utilizes bispectrum analysis, uses different topologies for convolutional neural networks to pull out features, and chooses robust features to classify protein families. The goal is to outperform existing methods for identifying protein families, thereby enhancing classification metrics. The materials consist of numerous protein datasets, whereas the methods incorporate bispectrum characteristics and deep learning strategies. The results of this study demonstrate that the proposed method for identifying protein families is superior to conventional approaches. Significantly enhanced quality metrics demonstrated the efficacy of the combined bispectrum and deep learning approaches. These findings have the potential to advance the field of protein biology and facilitate pharmaceutical innovation. In conclusion, this study presents a novel method that employs bispectrum characteristics and deep learning techniques to improve the precision and efficiency of protein family identification. The demonstrated advancements in classification metrics demonstrate this method’s applicability to numerous scientific disciplines. This furthers our understanding of protein function and its implications for disease and treatment.

## Section 1: Introduction

Nearly every cellular reaction and metabolic process in living organisms involves proteins. Proteins play crucial roles in biological and disease processes, but only if their structure, function, and interrelationships are understood [1,2]. Identifying protein families, or groups of proteins with similar structures and functions, is crucial to any protein investigation [3]. Recognizing protein families facilitates the comprehension of evolutionary relationships, the prediction of protein function, and the discovery of potential therapeutic targets [4]. Throughout the years, numerous classification methods for protein families have been developed. These techniques predominantly belong to the categories of traditional and contemporary computational approaches [5]. In this introductory section, the current state of the art in protein family identification, the limitations and challenges of the existing methods, and the need for a more efficient and accurate method will be investigated.

Historically, scientists have discovered novel protein families through biochemical assays, protein sequencing, and structural analysis. Analyzing vast protein collections was difficult, time-consuming, and beyond their capacity [6]. Due to the development of high-throughput technologies and the availability of large datasets of protein sequences, computational approaches have become practical tools for identifying protein families. By comparing protein sequences, sequence-based approaches identify commonalities and infer evolutionary relationships [7]. Using sequence alignment, the most prevalent sequence-based method, conserved regions, insertions, and deletions in protein sequences are identified. ClustalW (a multiple-sequence alignment program) and the Basic Local Alignment Search Tool (BLAST) are the most widely used alignment algorithms [8]. However, sequence-based methodologies have limitations, including the inability to identify distantly related proteins due to sequence divergence and the susceptibility to errors introduced by gaps and insertions. These techniques analyze the three-dimensional structure of proteins to identify similarities and common folding patterns. Protein structures are compared by structural alignment algorithms, such as DALI (Distance-matrix ALIgnment) and CE (Combinatorial Extension), based on the spatial arrangement of secondary structure elements [9]. The availability of empirically determined protein structures restricts the usefulness of structure-based methods for understanding protein function and evolution. Profile-based methods generate profiles or hidden Markov models (HMMs) from multiple sequence alignments of related proteins [10]. These profiles capture position-specific amino acid patterns (PSAAP) and protein family-wide conservation. HMMER (is a free and commonly used software package for sequence analysis) and Position-Specific Iterative BLAST (PSI-BLAST) are more sensitive than straightforward sequence alignment methods and can detect distant homologs [11]. However, profile-based methods necessitate a well-curated multiple-sequence alignment and can be computationally intensive.

Henceforth, identifying and classifying protein families is essential to understanding the complexities of protein structure, function, and evolutionary paths. In contrast, conventional approaches encounter challenges such as the necessity for enhanced computational speed, stringent criteria for database integrity, and susceptibility to sequence divergence. The emerging field of computational approaches, particularly those based on machine learning (ML) and deep learning (DL) paradigms, holds significant promise for overcoming these limitations. Combining the distinctive characteristics inherent to bispectrum analysis with the potent feature extraction capabilities innate to deep learning methodologies is the optimal strategy for enhancing the capacity for protein family identification. This combination could improve the precision and effectiveness of machine learning algorithms in this field. This innovative technology has the potential to enhance scientists’ understanding of protein biology and expedite the creation of novel treatment methods. This unique technology has the potential to enhance scientists’ understanding of protein biology and accelerate the development of new treatment approaches. In Section 2, the pertinent literature review is presented. In Section 3 of this study, methods for numerical encoding, bispectrum, and feature extraction using the most competent convolutional neural network (CNN) architectures, efficient feature selection methods, and the ML classification algorithm are discussed in detail. The results section elaborates on the algorithm’s execution and outputs and emphasizes inferences. Section 4 will discuss the outcomes and potential applications of the proposed technique. The study’s conclusions will explain the efficacy of the proposed method and identify areas that can be investigated further to enhance the accuracy of predictions.

## Section 2: Related works

An amino acid can be as a letter, a protein sequence as a library, and a motif as a paragraph. Insight into the physical structure’s functional qualities can be gained by exploring the relationships between these sequences. Scientists in biomolecular research are always looking for new ways to classify proteins based on their unique sets of amino acid residues. To this end, researchers classify sets of proteins with similar roles as "protein families”. However, uncharacterized proteins in different bioinformatics domains need to be identified and classified. Scientists typically represent groups of proteins with similar functions using a clustering motif. However, there is still a need for improvement in many areas of bioinformatics, such as protein identification and categorization. Therefore, a primary goal of applied research is to understand physicochemical processes [12].

Engineering-based techniques are needed to extract discrete or continuous features from protein sequences for classification purposes. Although traditional methods have significantly contributed to identifying protein families, they confront several challenges and limitations. The efficacy of these methods depends heavily on the quality and completeness of available protein sequence and structure databases. Inaccurate identification of protein families may result from incomplete or biased databases. Sequence divergence and structural variation make it difficult for conventional methodologies to identify distantly related proteins. This limitation hinders the comprehension of the evolution and function of proteins. Traditional methods are computationally intensive and may need to be more scalable to analyze the ever-increasing protein sequence data generated by high-throughput technologies [13]. Due to subjective parameter settings and assumptions made during the analysis, traditional methods may introduce biases and errors. Clustering and labeling tasks are prominent applications of unsupervised learning, a popular ML technique. Protein sequence pattern discovery is greatly aided by matching genetic characteristics to protein sequences. However, this motif comparison approach relies heavily on the knowledge of biological experts and subject-matter experts in order to identify functional motifs [14]. Before tinkering with a protein’s coding in the cell, its functionality in the body needs to be understood. One approach [15] for determining the total number of variables is to use a generalized series of Gaussian process regression. The precision and results of functional analysis can be improved through training on sequencing data from many proteins [16]. Researchers [17] used the Resonant Recognition Model (RRM) on the hormone Prolactin (PRL) to find resonant frequencies and predict "hot spots" in the protein sequence that are functionally important. The bulk of researchers’ recommended amino acids were compared to these findings. Initiating or altering biological processes, light wavelengths, and electromagnetic radiation have roles. The RRM postulates that infrared and visible light electromagnetic energy transmission is crucial to protein interactions. The RRM model applies spectral and space-frequency analysis to linear data, such as the linear sequences of components that make up proteins. When free electron energies interact with proteins, it regulates protein activity, and when molecules connect, it requires the transfer of electromagnetic energy between them at very particular frequencies [18]. Another study [19] tackled the sequence metric issue by performing multivariate statistical studies on numerous properties of amino acids. The study used factor analysis to calculate meaningful and understandable amino acid differences. This method makes it easier to analyze sequence data and produces ratings that can be used in other research [20]. The RRM and the Informational Spectrum Method (ISM) [21] were both detailed in the research. Two plasmodial peptides, P18 and P32, serve as illustrative examples of these processes, and their involvement has been explored using computational models [22]. Integer vectors can be fed into Support Vector Machine SVMs, decision trees, and machine-learning methods. Protein sequences can be encoded into numerical vectors with the "Protein Encoding" Matlab module [21], which was developed for bioinformatics research and features intuitive Matlab application programming interfaces (APIs). Autocorrelation descriptors, defined by the position-specific score matrix (PSSM)of evolutionary data along the amino acid sequence [22], can be utilized in addition to more traditional methods [12]. The PSSM, three autocorrelation descriptors, evolutionary and sequence-order data, and the resulting feature vector total 560 dimensions, making the model extremely detailed. The SVM classifier performs best when the 175 dominant features with the highest variance and lowest reconstruction error are utilized. Principal component analysis (PCA) is used to select features and minimize noise. The new model outperforms prior evolutionary information-based approaches, notably for amino acid sequences with low similarity, as shown by experimental findings from a Jackknife cross-validation test on three benchmark datasets [22].

A study [23] shows that amino acid codons map onto a complex prime number representation (CPNR). There are as many codons as there are prime numbers. This finding dramatically aids insight into the relationship between prime numbers and complex domain mapping. Numbers in CPNR are typically independent, meaning they cannot be created by performing arithmetic operations on a real number (such as adding, multiplying, or exponentiating) [24]. The study invistigated 520 protein sequences across seven different categories. Establishing a mathematical link between molecular structures and the behaviors under study is essential for constructing quantitative structure-activity relationships (QSARs) [25]. Molecular descriptors can be categorized using both experimental and theoretical descriptors. In [25], the authors provide an all-encompassing review of theoretical descriptors, molecular descriptor computation, and their numerous classifications and viewpoints. The research aimed to choose and model each amino acid index based on features like hydrophobicity and alpha to locate descriptors that yield more insightful protein modeling. Subsequently, [26] evaluated the possibility of physicochemical descriptors, the fast Fourier transform (FFT), and protein feature classification to improve prediction findings. Based on the information utilized to construct the code, encoding methods for amino acids are categorized into five groups: binary, physicochemical properties, evolution-based, structure-based, and machine learning. The research describes the five categories of amino acid encoding, discusses the proposed methodologies, and then examines sixteen approaches to encoding amino acids to ascertain protein shape and secondary structure [27]. Primary sequences alone can classify protein families and can be turned into mathematical representations of amino acid sequences [23]. Using the integer representation of amino acid codes, the study offers a mapping technique using Fibonacci numbers and a hashing table (FIBHASH). A Fibonacci number was ascribed to each numeric representation of an amino acid. These 20-byte hash tables were utilized to retain the amino acid codes fed into recurrent neural networks for grouping [28]. The classification of proteins is essential to both medical diagnosis and treatment. This level of accuracy could not have been attained with more conventional classification methods.

The results were significantly improved when machine learning and deep learning techniques were employed. All ML algorithms must convert protein sequences to numeric form, and if this is done flawlessly, performance can be enhanced [23,28]. Multiple aspects of protein sequences, including amino acid physiochemistry and three-dimensional structure, can be represented. Using this method, it is difficult to identify the optimal numerical representation for protein sequences. Researchers have studied two distinct encoding methodologies for mapping protein sequence-function relationships over the past decade. Using the conventional encoding method ("one-hot encoding"), the binary representation of an amino acid sequence is provided immediately. In a "learned encoding" scheme [29], millions of unlabeled protein sequences are used to train an unsupervised ML algorithm. The trained encoding method permits protein sequences encoding as numerical vector representations. To fulfill their biological function, proteins must interact in a particular manner, and the learned encoding scheme assumes that all protein sequences conform to the evolutionary principles or biophysical properties that govern these interactions [30]. The vector representations of the taught encoding method illustrate how proteins are related in the sequence space that has been learned. Similar vector representations can be expected for identical sequences when using downstream-supervised ML models, like the Gaussian process (GP) [31]. This model means that similar biological functions can be assumed using models like the GP.

Using CNNs to predict the secondary structure of proteins is a relatively recent application [32,33]. In [32], the prediction was based on the PSI-BLAST position-specific score matrix profile. In [33], the amino acid sequence properties were mixed with 1D kernel motions. In [34] they combined experimentally collected structural information of enzymes with deep learning techniques to construct models that predict enzymatic function based on structure. The article’s authors [23] developed a protein mapping technique to convert amino acid sequences into numerical representations, which they then used to predict protein families.

A bispectral analysis is a cutting-edge data processing technique that accounts for phase coupling (quadratic nonlinearities) between nonlinearly behaving signal components. Numerous biological signals, such as the electrocardiogram (ECG) and heart sounds, are distinct due to their interdependencies [35–38]. The additional data points these techniques provide may improve the performance of the deep learning system. Recent research [39] employed a hybrid bispectral deep neural network to classify ten families within the Globin-like superfamily. This procedure improved the categorization problem significantly in comparison to the previous ones. Despite these results, 16 families still required assistance [39]. Using numerically encoded bispectrum images of protein sequences and a well-designed two-stage CNN model classifier, [40] introduces a new method for identifying the 16 protein families that comprise the Globin-like superfamily. Consequently, a more efficient and accurate method of protein family identification is urgently required to resolve the limitations of existing approaches. Recent advancements in machine learning and deep learning have enabled the development of innovative computational methods. These methods allow more accurate identification of distant homologs and protein function prediction by utilizing large-scale protein sequence and structural data. When sequence, structural, and functional annotations are merged, it is easier to comprehend the relationships between protein families. Incorporating these revolutionary methodologies into user-friendly tools and software programs could accelerate the advancement of protein biology.

As such, the main obstacle in protein research pertains to the efficient identification of protein families to comprehensively understand their structural, functional, and evolutionary attributes. Although traditional methods have proven helpful, they are also associated with certain limitations. These limitations include computational inefficiency, strict requirements for database quality, and susceptibility to sequence divergence.

In order to overcome these constraints, the present work proposes an innovative methodology that leverages the capabilities of cutting-edge computational techniques, including machine learning and deep learning. This study seeks to address the current challenges in protein family identification by combining bispectrum features and deep learning feature optimization approaches. The primary contributions of this study are as follows:

1. The present study presents a novel approach to enhance efficiency in identifying protein families. This method enables expedited and comprehensive studies of protein datasets by mitigating computational complexity.
2. Using bispectrum characteristics and deep learning approaches enables extracting resilient and distinctive features from protein sequences, hence augmenting categorization accuracy.
3. Enhanced Precision: By utilizing exact machine learning algorithms, this innovative methodology can achieve improved outcomes in identifying protein families, thereby transcending the constraints of current methodologies.
4. Enhanced Comprehension: The suggested methodology enables researchers to explore protein biology at a more profound level, facilitating a deeper understanding of protein structure, function, and evolution.
5. The method of accelerated therapeutic development utilizes improvements in protein family identification to expedite the development of new medicines, hence providing significant benefits to scientific research and medical innovation.

## Section 3: Methodology

**Fig 1:**
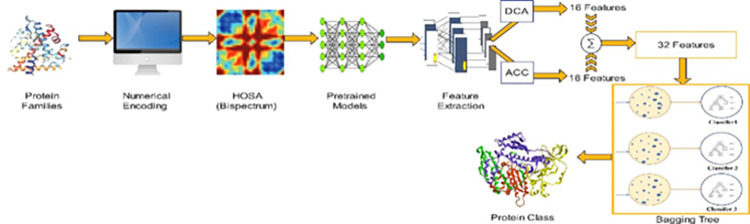
The proposed method.

Fig 1 depicts the proposed method in this paper, as shown, the procedure from encoding protein sequences, passing to higher order spectral representation (Bispectrum), then utilizing the pre-trained convolutional neural networks to extract the graphical features using the transfer learning techniques. In this paper, various features engineering algorithms are employed to enhance the performance of classification and obtain the best representative attributes among all extracted ones.

For more illustration the corresponding pseudocode was employed to obtained the best results

**# Step 1: Extract Features from the Last Fully Connected Layer of CNN**

Initialize CNN model

Load pre-trained weights

Modify the output layer to be compatible with 16 classes

# Extract features from a dataset of images (Split the dataset into 70% training, 30% testing)

for each image in the dataset:

 features = CNN.predict(image)

 store features to featureMatrix_Training

Store the test features in featureMatrix_Testing

#Repeat the same process for five pre-trained CNNs

**# Step 2: Apply Canonical Correlation Analysis (CCA) and Dimensionality Reduction (DCA)**

*# Apply CCA to the feature_list*

cca_features = apply_CCA(featureMatrix)

*# Apply DCA to CCA Feature*

dca_features = apply_DCA(cca_features)

**Step3: Combined 16 features from CCA, and 16 Features from DCA**

**# Step 4: Build Bagging Tree Ensemble Classifier with 70% of combined reduced features**

Initialize empty ensemble_list

*# Repeat for a specified number of iterations*

for each iteration:

 *# Randomly sample training data with replacement for each iteration*

 sampled_training_features, sampled_training_labels = bootstrap_sample(training_features, training_labels)

 *# Create and train a decision tree classifier on the sampled data*

 tree_classifier = create_and_train_decision_tree(sampled_training_features, sampled_training_labels)

*# Add the trained tree classifier to the ensemble*

 append tree_classifier to ensemble_list

**# Step 5: Classification with the Ensemble**

**# Initialize an array for storing predictions**

predictions = []

*# For each data point in testing_features*

 # Initialize an array to store predictions from each tree in the ensemble

 tree_predictions = []

 *# Make predictions using each tree in the ensemble*

 for each tree_classifier in ensemble_list:

  tree_prediction = tree_classifier.predict(data_point)

  append tree_prediction to tree_predictions

 # Calculate the aggregation method for tree predictions

 final_prediction = aggregate_predictions(tree_predictions)

 # Append the final prediction to the predictions array

 append final_prediction to predictions.

**# Step 6: Evaluate the Ensemble Classifier**

Calculate accuracy, precision, recall, F1-score, etc., for the predictions.

### Database

The present study uses the superfamily data obtained from the InterPro website, which is currently the location of the Pfam database. InterPro is a comprehensive repository of protein families, domains, and functional sites [41]. The comprehensive and unified nature of the superfamily data on the InterPro website, which currently hosts the Pfam database, is the main driving force behind its selection. *InterPro* is a central repository incorporating and harmonizing data from multiple protein signature databases, such as Pfam, PRINTS, PROSITE, and SMART. This integration presents several significant benefits: InterPro provides a more comprehensive view of protein superfamilies by incorporating data from multiple sources. Researchers have access to a more extensive variety of protein families and domains in a single location, eradicating the need to consult multiple databases independently. InterPro consolidates and standardizes data, facilitating researchers’ ability to navigate and interpret the information. This consistency expeditiously improves the dependability and comparability of information across numerous protein families and domains. The platform permits the cross-referencing and coupling of various protein families and domains. This interconnectedness allows researchers to investigate relationships and functional associations between various protein superfamilies, thereby augmenting the depth of their analyses. InterPro routinely updates and maintains its integrated databases, ensuring that researchers have access to the most current and accurate information. This function is essential for maintaining protein data evolution. InterPro provides a user-friendly interface for searching, retrieving, and visualizing information regarding protein superfamilies. Researchers have adequate access to the data they need to conduct investigations. Finally, InterPro promotes global research collaboration by encouraging participation and contributions from the community. This collaborative strategy improves the quantity and quality of data that is currently accessible. Furthermore, It facilitates the examination of protein sequences by leveraging their distinctive signatures, which are derived from prediction models like hidden Markov models. The capacity of InterPro to amalgamate the protein signatures originating from its constituent databases into a unified and exploratory repository is a paramount attribute. Furthermore, it has the potential to leverage the unique attributes of each database in order to construct a unified and resilient database and diagnostic tool [40].

### Encoding method

The precise encoding of amino acids plays a pivotal role in determining the overall efficacy of categorization methodologies. In stark contrast to the encoding of protein sequences, the encoding of amino acids employs a fusion of diverse methodologies to forecast the characteristics of a protein, encompassing both the individual residues and the overall sequence. Encoding methods are commonly classified into five distinct categories, determined by the origin of the information and how it is extracted. These categories include binary encoding, physicochemical characteristics encoding, evolution-based encoding, structure-based encoding, and machine-learning encoding. The article portrays amino acids within protein sequences as binary numbers with multiple dimensions, specifically 0 and 1. As mentioned above, the procedure is commonly referred to as a binary encoding technique [27].

The commonly employed terminology for the digital representation of amino acids includes feature extraction, amino acid encoding scheme, or residue encoding scheme [27]. One-hot encoding, also referred to as orthogonal encoding, is a widely utilized binary encoding technique [42]. In the context of the one-hot encoding method, it is observed that a binary vector with a dimensionality of twenty is utilized to represent each of the twenty standard amino acids. The precise arrangement of the twenty standard amino acids is explicitly delineated. The ith amino acid type is represented by employing a binary encoding scheme consisting of twenty bits. In this encoding, the ith bit is assigned a value of "1," while the remaining bits are assigned a value of "0." Every vector possesses a solitary binary digit, denoted by the symbol "1". Henceforth, it is referred to as "one-hot." The arrangement of the twenty standard amino acids is denoted as [A, C, D, E, F, G, H, I, K, L, M, N, P, Q, R, S, T, V, W, Y]. Each amino acid is assigned a one-hot code, wherein the one-hot code for A is 1000000000000000, the one-hot code for C is 0100000000000000, and so forth. Given the presence of unidentified amino acids within protein sequences, it is imperative to acknowledge that, under certain circumstances, an additional unit is necessary to denote the unidentified amino acid type [40]. Consequently, the binary vector’s length will extend to twenty-one, as stated in reference [27]. The classification accuracy of the utilized encoding method can be improved by normalizing its outputs. This normalization process involves utilizing the mean value and standard deviation of the encoded amino acid distribution within each family, as explained in [40].

### Bispectrum

The bispectral analysis is a scientifically rigorous signal processing technique investigating the intricate phase coupling between distinct signal components, explicitly focusing on the intricate interplay of values encapsulated within proteins. After a short explanation of how bispectral analysis works, CNN uses the insights gained from it to place proteins in their own families [43]. Nonlinearities are deviations from a straight line in the process of encoding proteins, and how nonstationarity is shown changes the connections between frequencies within these families. The bispectral analysis is a sophisticated signal processing technique that quantifies quadratic nonlinearities and deviations from linearity. It quantifies the interdependence of signal constituents, such as the representation encoding proteins. Modifications to the bispectrum can be quantifiably observed when there are alterations in the representation that lead to different quadratic nonlinearities. More information about the utilization of bispectrum is available in [40,43].

### Pre-trained models

#### Squeeze net

SqueezeNet is a deep neural network architecture created for efficient and lightweight image classification. It was designed by investigators at Deep Scale, Inc. and released in 2016. The primary purpose of SqueezeNet is to perform high accuracy on image classification tasks by optimizing the model size and the computation.

The key notion behind SqueezeNet is to remarkably diminish the number of parameters in the network by utilizing a variety of 1x1 convolutional filters, also known as "squeeze layers," and "expand layers." These layers help to reduce the computational burden while retaining good accuracy.

The 1x1 filters are employed in the squeeze layers to reduce the depth dimension of the input tensor, thus squeezing the information [44]. SqueezeNet has been achieved popularity in various applications with computational resources are limited; such as mobile and embedded devices. Its lightweight nature makes it appropriate for real-time image analysis on devices with limited processing power [45].

#### Shuffle net

Shuffle Net is an extremely computation-efficient CNN architecture, which is designed especially for mobile devices with very limited computing power (e.g., 10–150 MFLOPs). This architecture utilizes the pointwise group convolution and the channel shuffle to greatly reduce computation cost while maintaining accuracy. Table 1 displays the total ShuffleNet architecture. It consists of three stages made up of a stack of ShuffleNet units. The pointwise convolutions’ connection sparsity is controlled by the group number. The output channels can be computed and assessed simultaneously by assigning different values for g, ensuring that the overall computational costs are roughly the same (140 MFLOPs) [46].

**Table 1:** The results for the first eight families: Number of true positive, true negative, false positive, false negative, precision, sensitivity, specificity, and F1-secore for each class individually.

Evaluation Criteria	TP	FP	FN	TN	Precision	Sensitivity	Specificity	F1-Score
Family01	114	4	2	1736	97	98	100	97
Family02	101	22	14	1719	82	88	99	85
Family03	113	5	3	1735	96	97	100	97
Family04	107	12	7	1730	90	94	99	92
Family05	112	0	4	1740	100	97	100	98
Family06	98	18	18	1722	84	84	99	84
Family07	116	2	0	1738	98	100	100	99
Family08	108	10	8	1730	92	93	99	92
Family09	105	3	11	1737	97	91	100	94
Family10	101	11	15	1729	90	87	99	89
Family11	101	11	15	1729	90	87	99	89
Family12	113	1	3	1739	99	97	100	98
Family13	113	5	1	1735	96	99	100	97
Family14	115	4	1	1736	97	99	100	98
Family15	99	10	17	1730	91	85	99	88
Family16	108	2	8	1738	98	93	100	96

#### ResNet101

A residual learning framework makes it easier to train networks that are much deeper than those that were previously used by reformulating the layers so that they learn residual functions with reference to the layer inputs rather than learning unreferenced functions. It also provides extensive empirical evidence demonstrating that these residual networks are simpler to optimize and can gain accuracy from greatly increased depth [47].

**Fig 2:**
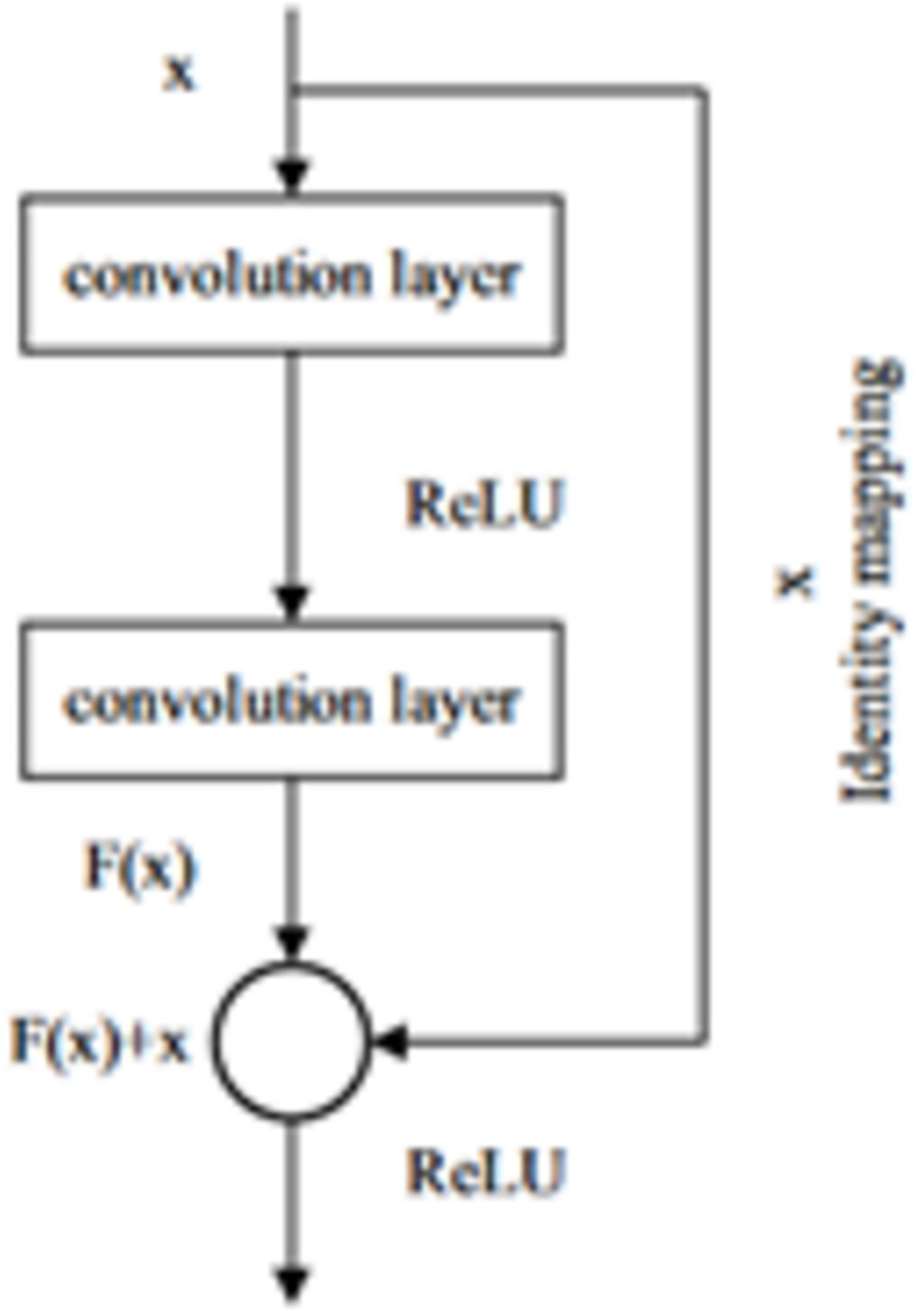
The structure of the residual block [49].

Wu et al.’s [48] proposal of a residual network to improve feature transmission by incorporating shortcut connections into the convolutional neural network was made in response to this issue. Every two layers of conventional convolution are followed by the addition of a short-cut to create residual blocks. A residual network is created by connecting several residual blocks. As seen in Figure below, x serves as the network’s input. The result of two convolution layers is represented by the function F(x). The original output will be superimposed with the mapping of quick connection F(x) + x before being sent to the following layer [49]. The structure of the layer is illustrated in Fig 2.

#### DarkNet-19

Darknet-19 is a new classification model used as the base of YOLOv2. The model is based on earlier research on network architecture as well as prevailing wisdom in the industry. We mostly employ 3 × 3 filters and increase the number of channels after each pooling phase, much like the VGG models [50]. In line with the research on Network in Network, predictions were made using global average pooling and the feature representation was compressed using 1 × 1 filters between 3 × 3 convolutions [51]. Also, to stabilize training, speed up convergence, and regularize the model used batch normalization [52]. The final model, called Darknet-19 has 19 convolutional layers and 5 max pooling layers [53].

#### NasNet

The Google ML group created the NASNet model in 2017 while researching new approaches to creating ConvNets. It is based on the Neural Architecture Search (NAS) method that used to find the best architectures based on gradients [54]. A CNN’s "Child Network" has a parent AI called a Recurrent Neural Network (RNN) namely "The Controller" that evaluates the effectiveness of the child AI and modifies the design of the "Child Network". The operational building blocks that the controller RNN may utilize to construct the "Child Network" are described in figure below. Adjustments are made to the number of layers, regularization techniques, weights, and other factors to increase the effectiveness of the "Child Network." [55]. NASNetLarge and NASNetMobile, are two distinct types of NASNet architectures, are created by training the architecture with two different picture sizes. Due to the difference in parameters between the two networks, NASNetmobile is significantly more dependable than NASNetLarge [54]. Every NASNet type has a block as its smallest unit. A cell is made up of a number of operational blocks, including those mentioned above, and it is made up of several cells that make up the NASNet architecture. Because the controller RNN optimizes the cells with blocks for a particular dataset, these cells are not fixed [55].

### Feature fusion

In recent network architectures, feature fusion—the combining of features from many levels or branches—is pervasive. It is frequently carried out by using straightforward operations like addition and concatenation, although this may not be the best option [56]. However, the performance of the created classifier may show the use of most representative features. Finding the most important characteristics is thus a significant challenge for computer-aided diagnosis systems [57]. This paper applies two types of fusion algorithms: CCA and DCA to classify protein families with highly accurate results.

### Canonical correlation analysis

Canonical correlation analysis (CCA) is a technique for comparing linear relationships between two variables with multiple dimensions. CCA can be thought of as using complicated labels to direct feature selection in the direction of the underlying semantics, the representation of the semantics is extracted by CCA using two perspectives of the same semantic object [58]. To extract cross-modal correlations, Deep CCA, based on the encoder-decoder network, maximizes the significance of multimodal data. Furthermore, the canonical projective vectors in the traditional CCA method comply with conjugated orthogonality criteria, making CCA a crucial technique for the extraction and fusion of numerous features. Examples of real applications contain little class information, although class knowledge is useful for CCA [59].

**Fig 3:**
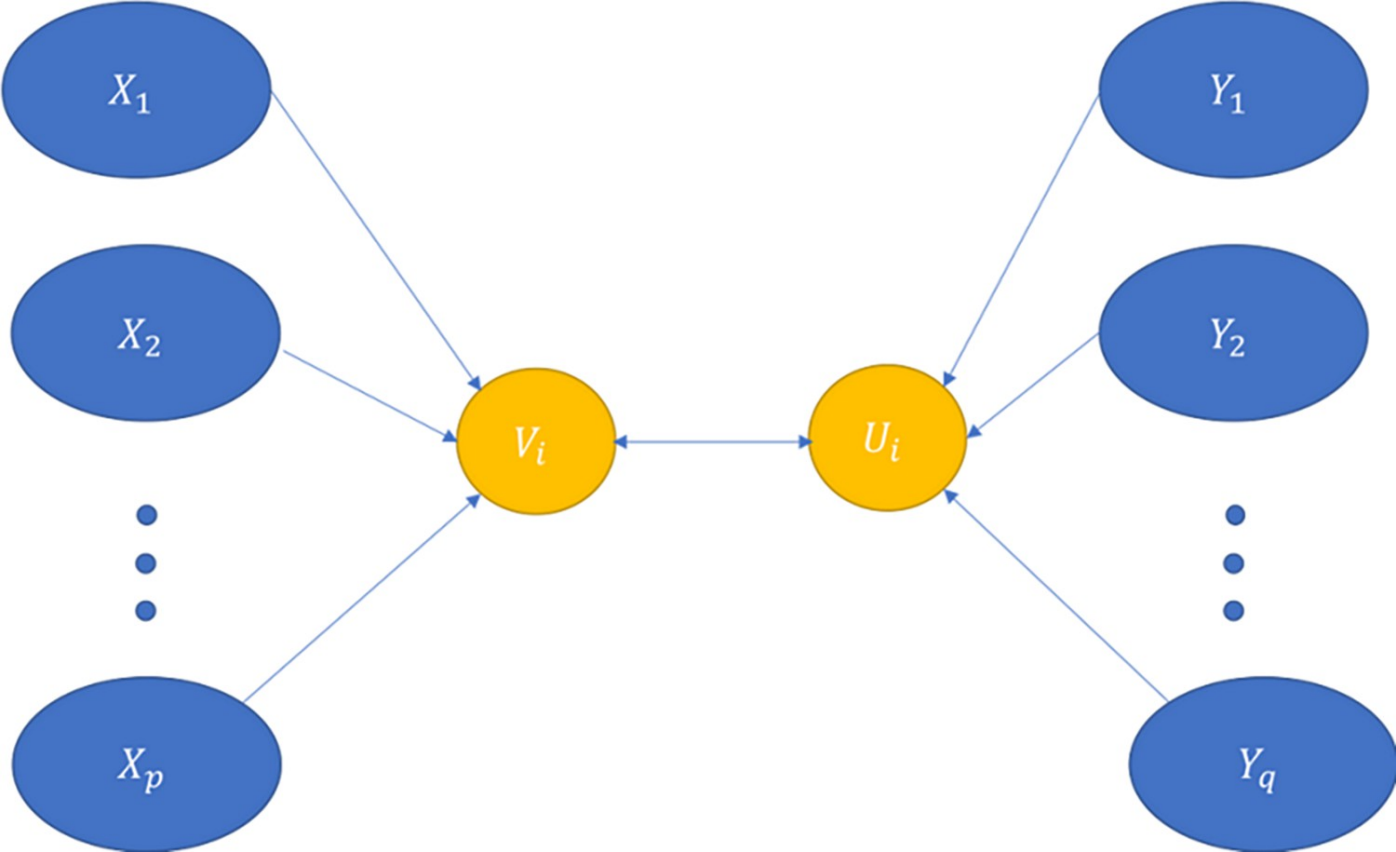
Components of a CCA function.

As it can be viewed in the Fig 3, it shows sets of variables X, Y, and the number of independent and dependent variables are p and q, respectively. All variables X and Y are lumped into two different variables, shown as yellowish circles in the figure. CCA aims to find the relationship between two lumped variables in a way that the correlation between these two is maximum. There are several linear combinations of variables, but the aim is to pick only those linear functions which best express the correlations between the two variable sets. These linear functions are called canonical variables, and the correlations between corresponding pairs of canonical variables are called canonical correlations.

### Discriminant correlation analysis

In biometric recognition, multiple types of features provide richer and more complementary information, making feature fusion an essential topic of research. Discriminant correlation analysis (DCA) is a feature-level fusion technique that includes the class relationships in the correlation analysis of the feature sets. By maximizing the pairwise correlations between the two feature sets, removing the correlations across classes, and only allowing correlations within the classes, DCA achieves an efficient feature fusion [60,61].

### Machine learning classifier

Machine learning employs an important role in the detection and classification of various applications in medical fields. In this paper, a bootstrap aggregating classifier is exploited to discriminate between 16 types of protein families. Bootstrap aggregating is well known as a bagging ensemble classifier, which is commonly used in decision tree algorithms. The bootstrap depends mainly on the selection of samples from the training dataset randomly with replacement, where, is the number of whole training samples. Each sample is used to build a classifier model. All models are utilized to predict the test samples based on majority voting of all aggregating models [62]. During training, decision trees learn multiple splits at each node. Surrogate splits are the next-best splits that help estimate the behavior of the primary split for those data points where the principal split isn’t appropriate [63].

In this paper, the number of learning cycles is 50 and surrogate splitting is utilized to split the trees.

For each input x, Bagging combines the predictions from individual decision trees and selects the class label with the highest aggregated count or probability. Soft averaging is performed in this paper where each tree in the ensemble outputs class probabilities, and the final prediction is determined by averaging the probabilities. The class with the highest average probability is selected as the final prediction. Mathematically, it can be represented as [64]:

$$\hat{y}=argmax_{c}(\frac{1}{N}\overset{N}{\underset{i=1}{\sum }}P(h_{i}(x)=c)$$

N is the number of trees in the ensemble, in our paper is equal to the number of training data.

$\left(P(h_{i}(x)=c\right)$ represents the probability assigned by the i-th tree to class c for input x.

The final prediction $\hat{y}$ is the class that has the highest average probability across all trees.

## Section 4: Results & discussion

The Convolutional Neural Networks (CNNs) field includes a wide range of architectural designs, each with its own configuration parameters, such as the number of layers, the size of the filters, the length of the stride, and other hyperparameters. These architectural variances inherently engender the extraction of a wide array of features. One can access a broader spectrum of features using multiple CNN models, which is potentially advantageous for the targeted task at hand. Each CNN model possesses the capacity to excel in capturing specific categories of features or discerning particular patterns within the data. To illustrate, specific models may exhibit proficiency in discerning intricate, fine-grained details, whereas others might specialize in capturing higher-level, semantically rich information. The amalgamation of features derived from multiple CNN models allows for a more all-encompassing and holistic data representation. Ensembling, which entails amalgamating predictions or features generated by multiple models, is a well-established technique for enhancing model performance. One can employ ensembling methods to produce a more resilient and precise data representation by extracting features from diverse CNN models. This approach mitigates the risk of overfitting and curtailing model variance, ultimately contributing to improved model robustness.

**Fig 4:**
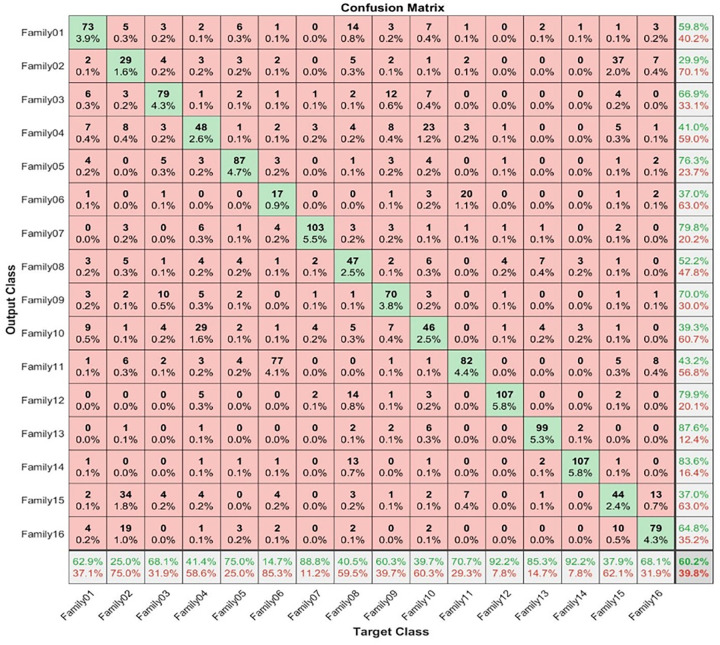
ResNet-101 confusion matrix.

The resultant images for all protein sequence families are processed to the five pre-mentioned pre-trained deep learning structures ResNet-101, Shuffle Net, NasNet, DarkNet, and SqueezeNet. Transfer learning is performed on the last fully connected layer to obtain the same number of intended classes. The classification of sixteen families using deep learning only was not efficient, the accuracy was very low. One of the pretrained model is illustrated in Fig 4. The accuracy achieved using ResNet-101 did not exceed 60%.

To enhance the prediction results for protein sequence, transfer learning is performed by replacing the last fully connected layer with a new one to obtain the same number of intended classes while other layers are unchanged. The specifications that have been used in all pre-trained models are the optimizer is RMSProp, the mini batch size is 128, the number of epochs is 20 and the learning rate is 0.01.

Beside to the data is divided into 70% training to train all models and extract and the training features and 30% for testing to extract the text features, as well.

Each network deserves 16 features, therefore, the total extracted features from five pre-trained models is 80. Feature fusion algorithms are applied either using CCA or DCA. In each stage, eighty features are passed, and the best sixteen features are selected. The resultant features from each stage are merged to obtain the best 32 features from all pretrained models and feature fusion techniques. One of the most popular machine learning classifiers; the bagging tree is exploited to obtain the best results. The extracted features from both training and testing sets were subjected to various experiments. The initial experiment concentrated on using deep learning as a feature extractor, involving the utilization of features derived from either the test cases or the training cases. These extracted features were then divided into 70% as training to build the machine learning model and 30% for testing.

Another scenario involved splitting the available features into three parts: 70% for training, 10% for validation, and 20% for testing.

In the final scenario, the approach’s validity was ensured by using the training features obtained from deep learning as attributes for constructing the machine learning classifier, and the model was tested using the features extracted during the deep learning stage.

**Fig 5:**
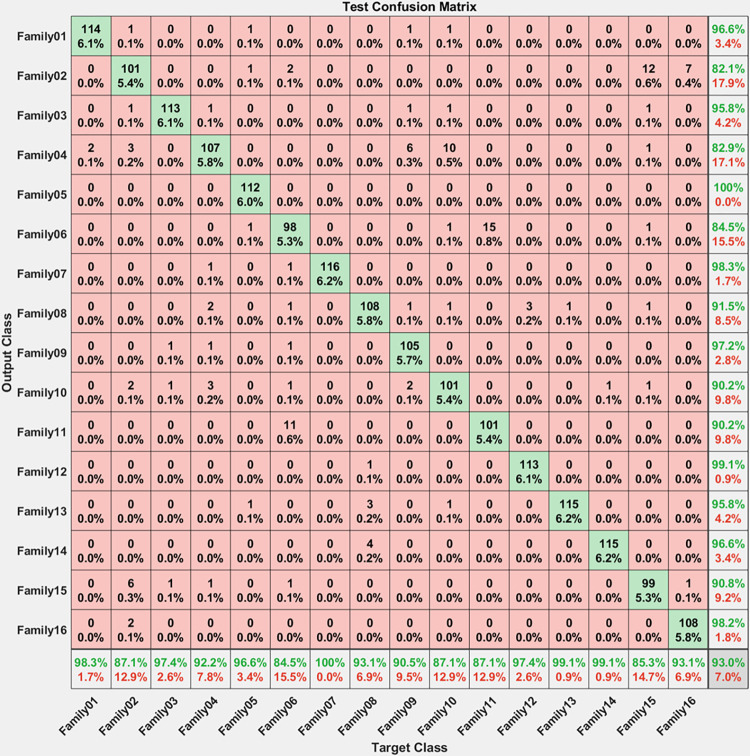
Confusion matrix for 30% testing.

All the features employed in these scenarios underwent feature selection techniques before being used. Fig 5 depicts the confusion matrix for testing the first scenario with 30% testing.

The evaluation metrics has been used in this paper are described by the corresponding equation [59].


$$Accuracy=\frac{TP+TN}{TP+TN+FP+FN}$$

$$Sensitivity=\frac{TP}{TP+FN}$$

$$Precision=\frac{TP}{TP+FP}$$

$$Specifcity=\frac{TN}{TN+FP}$$

$$F1-score=\frac{2\times Precision\times Sensitivity}{Precision+Sensitivity}$$

TP indicates to positive correctly classified cases for the class. TN represents the negative correctly classified cases. FN describes to negative misclassified cases and FP refers to positive misclassified cases.

The corresponding confusion matrix shown in Fig 5 clarifies the performance of the proposed procedure. Sixteen families are recognized using the proposed hybrid approach. From Fig 4, 144 sequences are distinguished from 114 in Family 1, with a sensitivity that does not exceed 98.3% and a precision of 96.6%. Family 2 has a lower sensitivity of 87.1% for 101 correctly classified sequences out of 116. Their precision is 82.1%. However, Family 3 performs the worst of all protein families, with only 113 of 116 sequences correctly separated, having a precision of 95.8% and a sensitivity of 97.4%. Type 4 is the worst discriminated family, with a sensitivity of 92.9% and a precision of 82.2%. In contrast, 112 of the 116 cases identified in Family 5 are correctly classified. The sensitivity is 96.6%, and the best precision is 100%. The hybrid model distinguishes Family 6 from all families. Its output is 98 correct cases out of 116, with a sensitivity of 84.5% and a precision of 84.5%. The proposed method attempts to distinguish Family 7 with 100% sensitivity and 98.3% precision. Family 8 performs better, with a true positive rate of 93.1% and a positive predictive value of 91.5%. Family 9 has a sensitivity of 90.5% and 97.2% as precision. Whereas class 10 have almost identical results regarding precision. Almost 11 cases from family 6 are classified as class 11 with a positive predictive value of 90.52% and a true positive rate of 87.1%. On the other hand, only 1 sequence from family 8 is classified as class 12 with a precision of 99.1%. However, 3 protein sequences from category 12 are misclassified as family 8. The sensitivity of family 13 is 99.1% and precision is 95.8%. The recall is 99.1% for class 14 with 115 correctly classified from 116. For family 15 only 99 cases are classified correctly from 116 with a recall value of 85.3% and a positive predictive value of 90.8%. For class 16, only 108 sequences are classified correctly from 116 with a recall of 93.1% and precision of 98.2%. The test accuracy of the proposed system is 93%. Table 1 summarizes the results obtained using the proposed method regarding the number of true positives, true negatives, false positives, and false negatives, as well as precision, sensitivity, specificity, and F1-score for each class individually.

**Fig 6:**
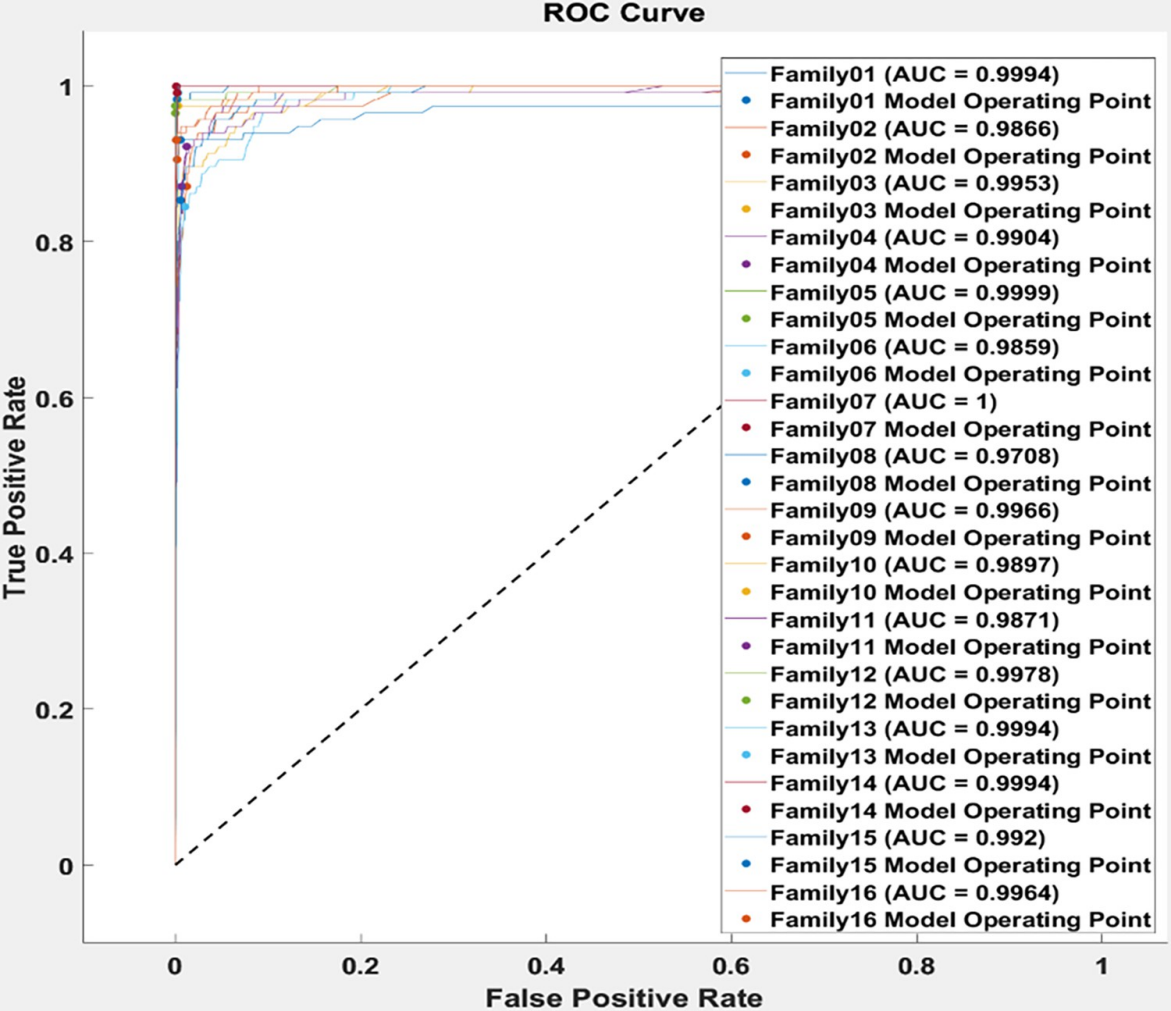
The receiver operating characteristics (ROC) for each class.

The performance of the proposed method is evaluated utilizing the receiver operating characteristic curve which describes the relation between the true positive rate on the y-axis versus the false positive rate on the x-axis. That leads to the area under the curve (AUC) for each class separately. As the AUC is almost 1, that refers to the system being sensitive to positive cases. Fig 6 implies the AUC for sixteen families. It depicts that the proposed approach reaches almost one AUC for all classes. As clear from ROC curve the proposed approach achieved the highest area under the curve for all protein family sequences, it is almost 1 in all cases. That reveals the ability of the proposed model to classify the protein family using distinguished features and without needing for further models.

**Fig 7:**
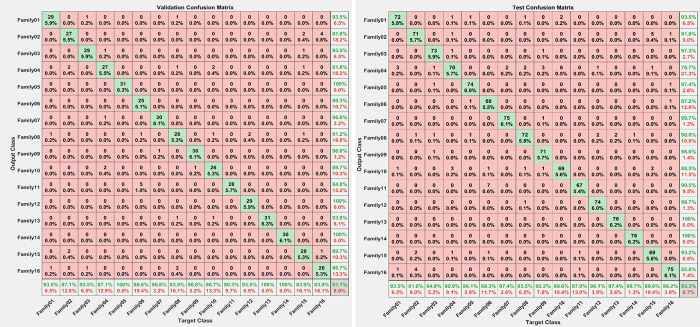
Confusion matrices results for second scenario (a) Validation Confusion matrix, (b)Testing Confusion matrix.

Due to big dataset that has been used in this paper, holdout validation method is used by utilizing 70% training, 10%validation and 20% for testing. The corresponding confusion metrices represent the obtained results for both validation and testing confusion matrices in Fig 7(A) and 7(B), respectively.

The validation accuracy is 91.1%, and the testing accuracy is 93.3%. That can be interpretated as the bagging tree classifier depends mainly on creating multiple bootstrap samples from the training data to train individual decision trees on these samples. Therefore, each tree is slightly different due to the randomness in the bootstrapping method. By averaging the predictions of trees, the ensemble’s performance can be slightly better on the test set compared to the validation case due to the explained randomness. The other reason may come from data splitting, where the test set is more representative than the validation test, which causes the validation accuracy be slightly less than the test accuracy, as in our case.

The explanation of both confusion matrices is appeared in Tables 2 and 3. The all entries are, the number of true positive case, The number of false positive case, the number of false negative case, and the number of true negative cases, as well. The evaluations criteria are calculated as precision, sensitivity, specificity, and F1-score.

**Table 2:** Validation results.

Evaluation Criteria	TP	FP	FN	TN	Precision	Sensitivity	Specificity	F1-Score
Family01	29	2	2	461	94	94	100	94
Family02	27	4	4	459	87	87	99	87
Family03	29	2	2	461	94	94	100	94
Family04	27	6	4	457	82	87	99	84
Family05	31	0	0	463	100	100	100	100
Family06	25	3	6	460	89	81	99	85
Family07	30	1	1	462	97	97	100	97
Family08	26	6	5	457	81	84	99	83
Family09	30	1	1	462	97	97	100	97
Family10	26	3	4	461	90	87	99	88
Family11	28	0	3	463	100	90	100	95
Family12	29	0	2	463	100	94	100	97
Family13	31	2	0	461	94	100	100	97
Family14	30	0	0	464	100	100	100	100
Family15	26	3	5	460	90	84	99	87
Family16	26	4	5	459	87	84	99	85

**Table 3:** Test results.

Evaluation Criteria	TP	FP	FN	TN	Precision	Sensitivity	Specificity	F1-Score
Family01	72	5	5	1153	94	94	100	94
Family02	71	7	7	1150	91	91	99	91
Family03	73	2	4	1156	97	95	100	96
Family04	70	19	7	1139	79	91	98	84
Family05	74	2	3	1156	97	96	100	97
Family06	68	10	9	1148	87	88	99	88
Family07	75	1	4	1155	99	95	100	97
Family08	72	8	5	1150	90	94	99	92
Family09	71	1	6	1157	99	92	100	95
Family10	69	9	8	1149	88	90	99	89
Family11	67	7	10	1151	91	87	99	89
Family12	74	1	3	1157	99	96	100	97
Family13	76	0	2	1157	100	97	100	99
Family14	76	0	1	1158	100	99	100	99
Family15	69	5	1	1160	93	99	100	96
Family16	75	6	3	1151	93	96	99	94

**Fig 8:**
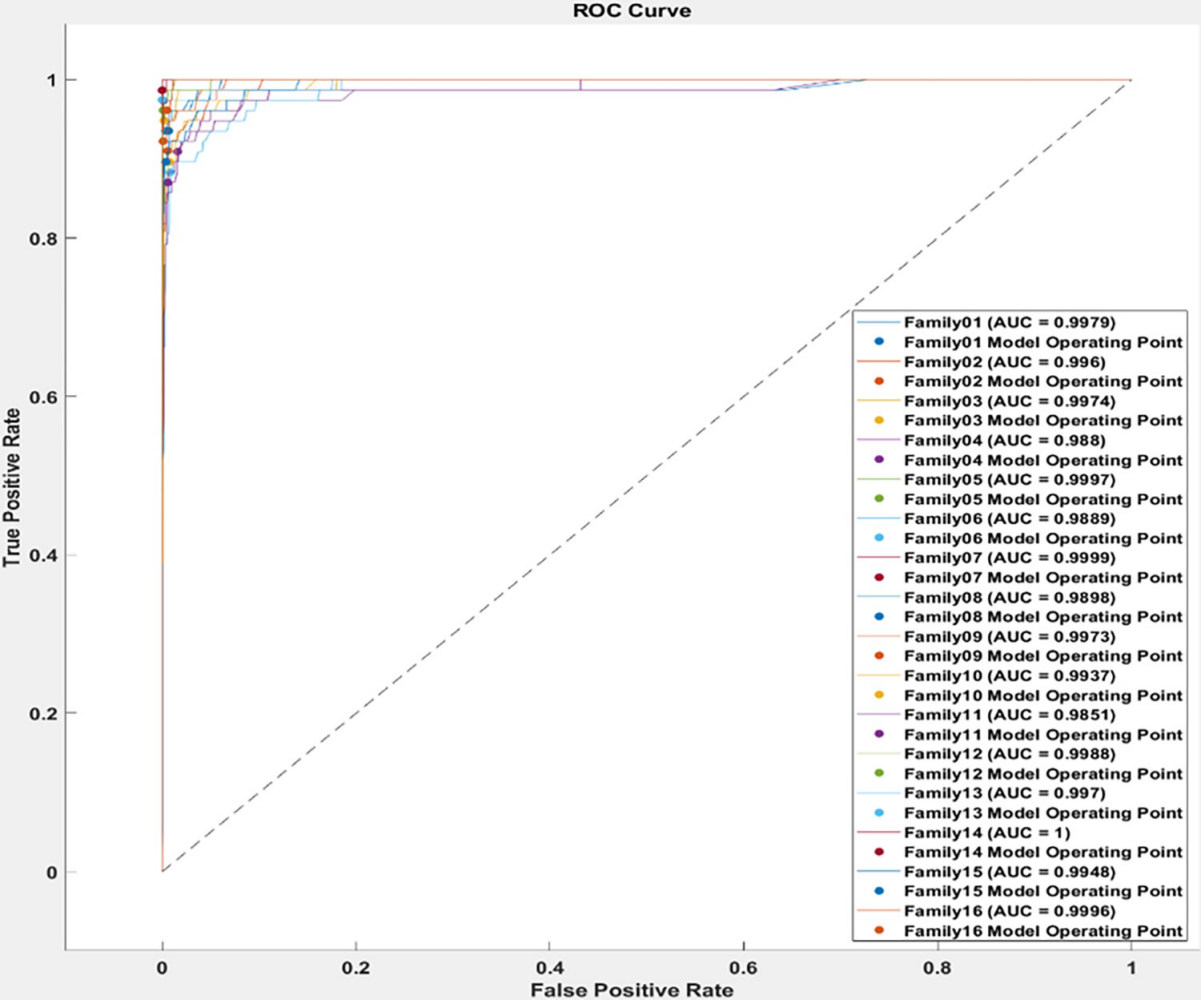
The ROC curve for the second scenario.

The ROC curve is depicted in Fig 8 for test cases. The AUC is almost 1 for all classes. The proposed system performs well in distinguish various protein sequences.

**Fig 9:**
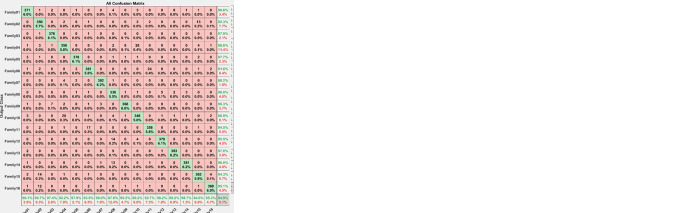
The confusion matrices for the third scenario (a) training, (b) test, (c) overall.

The third scenario is performed by keeping the reduced training features for building a bagging tree classifier and testing the model with the reduced test features. The training accuracy reached 98.6% for 16 classes and the test accuracy reached 80% with an overall accuracy of 94.6%. That indicates that the approach is valid and can be improved by using more represented methods for protein sequences, the confusion matrices are illustrated in Fig 9. That will be the future work.

The research examined the identification of protein families within the superfamily and its implications for advancing protein research. It highlighted the prospective impact of this discovery on drug discovery, disease treatment, and biotechnology. By correctly identifying protein families, researchers can obtain a deeper understanding of protein structure, function, and evolution, thereby facilitating the development of new drugs, targeted therapies, and advances in biotechnology. The study recommended combining bispectrum analysis with deep learning techniques to extract and select optimal features. It is proposed that the accuracy of protein family identification can be improved by employing a convolutional neural network (CNN) architecture and efficient feature selection methods. The research also indicated that support vector machine (Bagging Tree) classification is an efficient machine-learning technique.

The research highlighted the importance of evaluating the scalability of the proposed method on massive protein databases. As the quantity of protein sequence data generated by high-throughput technologies increases, this evaluation will assist in determining its effectiveness and efficiency in managing these data. Integration of multi-modal data, such as sequence, structure, and functional annotations, was also suggested to better comprehend protein families and their connections. In addition, the study highlighted the importance of user-friendly software tools and applications for implementing the suggested strategies. Such resources would expedite the discovery of protein biology and facilitate the efficient exploration of protein families. In order to test its efficacy and discover its distinctive contributions to protein family identification, the study suggests evaluating and comparing the suggested method to other state-of-the-art methodologies.

In the future, other approaches may be utilized as shown in [65]. An optimization problem with conflicting fault tolerance (FT) and communication delay objectives is created. Optimization is solved using an adapted non-dominated sorting-based genetic algorithm (A-NSGA). A-NSGA includes chromosome representation, FT and delays computation, crossover and mutation, and non-dominance-based sorting. Comparisons of performance were made using analytical and simulation methods. For further statistical analysis, [66], a multi-objective differential evolution variation with an improved mutation method solves the fundamental problem. The devised technique converges faster than others for many benchmark tasks. Finally, this algorithm finds the ideal temperature trajectories and OOC that counter heater malfunction.

The significance of identifying protein families within the superfamily and their potential implications for drug development, disease treatment, and biotechnology was investigated. It was suggested to increase precision by employing bispectrum analysis, deep learning methods, and compelling feature selection strategies. Future proposals for research and development should emphasize scalability, multi-modal data integration, the construction of user-friendly software tools, and comparative analyses of alternative methodologies.

## Section 5: Conclusions and future work

This research presents a comprehensive framework for advanced Protein classification and function prediction through the synergistic integration of bispectral analysis, machine learning, and deep learning. Protein classification and function prediction are essential steps in comprehending protein structure, function, and evolution, necessitating the assignment of proteins to their respective families. While conventional methods have made substantial progress in this regard, there remains a need for precision, scalability, and resistance to sequence divergence enhancements.

The proposed method, which leverages bispectral characteristics and deep learning techniques, enhances the identification of protein families. This work establishes a robust framework for classifying protein families through the amalgamation of numerical encoding, bispectrum analysis, convolutional neural network architectures, and feature selection techniques. The results affirm the viability of this strategy for applications in protein biology studies and drug discovery.

Future directions in protein family identification research should address several critical facets. First, the scalability of the proposed method warrants evaluation on large-scale protein datasets to gauge its efficacy and efficiency, a crucial consideration given the burgeoning volume of protein sequence data generated by high-throughput technologies. Second, incorporating multi-modal data encompassing sequence, structure, and functional annotations promises a more comprehensive understanding of protein families and their interrelations, augmenting the precision and depth of protein family identification. Additionally, the development of user-friendly software tools and products is imperative to facilitate the scientific community’s widespread adoption of advanced computational methods. Such tools will empower researchers to explore the realm of protein families more effectively, expediting discoveries in protein biology. In conclusion, rigorous evaluation and comparative analysis of our proposed method against contemporary techniques will further validate its efficacy and underscore its distinctive contributions to protein family identification. Research in these domains will propel our comprehension of protein biology, laying the foundation for innovative therapeutic interventions and drug discovery breakthroughs.
